# Soft wheat cultivars grown in the Saratov region and their resistance to Septoria blotch

**DOI:** 10.18699/VJGB-23-70

**Published:** 2023-10

**Authors:** Yu.V. Zeleneva, E.А. Konkova

**Affiliations:** All-Russian Research Institute of Plant Protection, Pushkin, St. Petersburg, Russia; Federal Center of Agriculture Research of the South-East Region, Saratov, Russia

**Keywords:** effector genes, PCR-diagnosis, wheat selection, Septoria blotch, phytopathogenic fungi, PtrToxA, PtrTox1, PtrTox3, гены-эффекторы, ПЦР-диагностика, селекция пшеницы, септориозы, фитопатогенные грибы, PtrToxA, PtrTox1, PtrTox3

## Abstract

Septoria is one of the harmful diseases of wheat cultivars cultivated in the Saratov region. This infectious disease of fungal etiology limits yield indicators and rapidly progresses in many regions of the Russian Federation. The aim of the research was to assess the resistance of winter and spring wheat cultivars that are referred to as promising and recommended for cultivation in the Low Volga region of the Russian Federation to pathogens of Septoria, to study the populations of Parastagonospora nodorum and P. pseudonodorum in the territory of the Saratov region in order to detect the presence of effector genes. Using molecular markers, we performed the identification of genes encoding NEs in 220 Parastagonospora spp. fungal isolates obtained from 7 cultivars of soft winter wheat, 6 taken from the winter triticale, 5 from soft spring wheat, 3 from durum spring wheat and 1 from spring oats. Among the P. nodorum isolates studied, there were both single genes Tox1, Tox3, and ToxA, and combinations of two genes in one genotype. The presence of the ToxA gene was not noted in the genotype of P. pseudonodorum isolates. During 2020–2022, a collection of winter and spring wheat cultivars was studied to detect resistance to Septoria blotch in field conditions (13 cultivars of winter wheat and 7 cultivars of spring wheat accordingly). The resistance of the cultivars was proven by laboratory evaluation. Three inoculums were used, including the isolates of Z. tritici, P. nodorum (ToxA, Tox1, Tox3), P. pseudonodorum (ToxA, Tox1, Tox3) mainly obtained from Saratov populations of 2022 (except for P. pseudonodorum with the ToxA gene). The tested cultivars were characterized using the Xfcp623 molecular marker, diagnostic for Tsn1/ tsn1 genes, which controls sensitivity to the fungal toxin of PtrToxA. Of greatest interest are 11 wheat genotypes that showed resistance to one, two and three species which served as causative agents of Septoria blotch (Zymoseptoria tritici, P. nodorum, P. pseudonodorum). These are the soft winter wheat cultivars Gostianum 237 (tsn1), Lutescens 230 (Tsn1), Guberniya (Tsn1), Podruga (Tsn1), Anastasia (Tsn1), Sosedka (Tsn1) and the soft spring wheat cultivars Favorit (tsn1), Prokhorovka (tsn1), Saratovskaya 70 (tsn1), Saratovskaya 73 (tsn1), Belyanka (tsn1). The results obtained are of interest as they might increase the efficiency of selection based on the elimination of genotypes with dominant Tsn1 alleles sensitive to PtrToxA. In addition to the economic value of the cultivars studied, it is recommended to use them in breeding for resistance to Septoria blotch.

## Abstract (ru)

Септориоз – одна из вредоносных болезней сортов пшеницы, возделываемых на территории Саратовской области. Это инфекционное заболевание грибной этиологии лимитирует показатели урожайности и быстро прогрессирует во многих регионах Российской Федерации. Целью исследований было оценить устой- чивость перспективных и рекомендуемых для возделывания на территории Нижневолжского региона РФ сортов озимой и яровой мягкой пшеницы к возбудителям септориозных пятнистостей и изучить популяции Parastagonospora nodorum и P. pseudonodorum, распространенных на территории Саратовской области, по наличию генов-эффекторов. C применением молекулярных маркеров проведена идентификация генов, кодирующих некротрофные эффекторы (NEs), у 220 изолятов гриба Parastagonospora spp., полученных с сортообразцов ози- мой и яровой мягкой пшеницы, яровой твердой пшеницы, озимого тритикале и ярового овса. Среди изученных изолятов P. nodorum были как единичные гены Tox1, Tox3 и ToxА, так и сочетания из двух генов в одном генотипе. В генотипе изолятов P. pseudonodorum не отмечено присутствие гена ToxА. Изучено 20 сортов озимой и яровой пшеницы на устойчивость к септориозным пятнистостям в лабораторных условиях и в поле в течение 2020– 2022 гг. Было использовано три инокулюма, включающих изоляты Zymoseptoria tritici, P. nodorum (TохА, Tох1, Tох3) и P. pseudonodorum (TохА, Tох1, Tох3). Анализируемые сорта были охарактеризованы с помощью молекулярно- го маркера Xfcp623, диагностического для генов Tsn1/tsn1, контролирующего чувствительность к токсину гриба PtrToxA. Наибольший интерес представляют 11 генотипов пшеницы, которые показали устойчивость к одному, двум и трем видам – возбудителям септориоза (Z. tritici, P. nodorum, P. pseudonodorum). Это сорта озимой мягкой пшеницы: Гостианум 237 (tsn1), Лютесценс 230 (Tsn1), Губерния (Tsn1), Подруга (Tsn1), Анастасия (Tsn1), Соседка (Tsn1) и яровой мягкой пшеницы: Фаворит (tsn1), Прохоровка (tsn1), Саратовская 70 (tsn1), Саратовская 73 (tsn1), Белянка (tsn1). Полученные результаты важны для повышения эффективности селекции на основе элиминации генотипов с доминантными аллелями Tsn1, чувствительными к грибу PtrToxA. Помимо хозяйственной ценности изученных сортов, их рекомендуется использовать в селекции на устойчивость к септориозной пятнистости

## Introduction

The Saratov region is a large administrative district; it includes 37 municipal districts, distributed based on climatic conditions between the right-bank black soil and arid steppe regions on the left bank of Volga. Differences in climatic conditions affect the yield of agricultural crops and the damage caused by diseases, among which the dominant position in the phytopathogenic complex is given to Septoria and pyrenophorous wheat blotch. During the years of epiphytoties caused by Septoria blotch in different regions of Russia, North America, Australia and other parts of the world, crop losses can exceed 30–40 % (Ficke et al., 2018; Sanin et al., 2018).

The annual monitoring implemented shows that in recent years in many regions of Russia, including the Saratov region, the pathogenic complex of wheat Septoria blotch has been dominated by the species of Zymoseptoria tritici (Desm.) Quaedvl. et Crous, representing the causative agent of Septoria leaf blotch on wheat, triticale, barley and rye (Zeleneva et al., 2022).

Less notable species are Parastagonospora nodorum (Berk.) Quaedvl., Verkley et Crous. They parasitize leaves, stems, glumes, awns of wheat and other cereals (Pakholkova, 2003; Sanin et al., 2018; Zeleneva et al., 2022). It quite often affects the seeds as well. The caryopsis turns puny, defective, its germination rate decreases. In case of strong infection, the coleoptiles get damaged and die off.

Another species, P. pseudonodorum, has a strict host specialization. It is a wheat parasite. Until recently, this species was considered a wheat form of P. avenae (A.B. Frank) Quaedvl., Verkley et Crous: P. avenae f. sp. triticea. However, based on the study of morphology using the methods of multilocus phylogeny in modern systematics, the species of P. pseudonodorum is one of the seven that have been described so far. In total, 26 species of Parastagonospora have been detected by now using phylogenetic analysis (Croll et al., 2021).

The fungi of P. nodorum and P. pseudonodorum are known for their ability to synthesize necrotrophic effectors (NEs), including host selective toxins (HSTs), that function as pathogenicity factors (Ciuffetti et al., 1997). Sensitivity towards NEs does not always lead to susceptibility of wheat to a Septoria pathogen (van Schie, Takken, 2014; Virdi et al., 2016).

Today, in total there are nine characterized interactions within the pathosystem of wheat – P. nodorum: Tsn1 – SnToxA (Friesen et al., 2009; Zhang et al., 2009; Faris et al., 2011); Snn1 – SnTox1 (Shi et al., 2016b); Snn2 – SnTox267 (Richards et al., 2022); Snn3-B1 – SnTox3 (Shi et al., 2016a); Snn3-D1 – SnTox3 (Zhang et al., 2011); Snn4 – SnTox4 (Abeysekara et al., 2012); Snn5 – SnTox5 (Sharma, 2019; Kariyawasam et al., 2022); Snn6 – SnTox267 (Richards et al., 2022); Snn7 – SnTox267 (Richards et al., 2022). It has been shown that genes encoding SnToxA, SnTox1, SnTox3 proteins are present in the genotype of P. pseudonodorum (Hafez et al., 2020; Navathe et al., 2020).

Right now there are three cloned host genes, including Tsn1 (Faris et al., 2010), Snn1 (Shi et al., 2016b) and Snn3- D1 (Zhang et al., 2021). There are also five fungus genes that encode effector proteins: SnToxA (Friesen et al., 2009), SnTox3 (Liu et al., 2009), SnTox1 (Liu et al., 2012), SnTox5 (Kariyawasam et al., 2022), and SnTox267 (Richards et al., 2022).

The increase in the aggression of crop culture fungal diseases was remarked in the Saratov region during the last decade. That is why the continuous search and use of new effective genetic sources and donors was and still remains the prioritized approach in wheat immunity selection in the Low Volga region (Konkova et al., 2022). The present research is annually conducted in the territory of the base provided by the Federal agrarian scientific centre of the South-East (in the city of Saratov). Starting from 2021, the selection of sources and donors expressing susceptibility to Septoria blotch has been conducted using molecular technologies that allow to pick genotypes with specific gene combinations.

The aim of the given research is to assess the resistance of soft winter and spring wheat cultivars recommended for cultivation in the territory of the Lower Volga region of the Russian Federation to Septoria blotch pathogens together with studying the populations of P. nodorum and P. pseudonodorum spread in the Saratov region territory to detect the presence of effector genes.

## Materials and methods

The affected plant samples were collected in 2021–2022 in the Saratov region territory. An infectious sample was understood as plant leaves with well-pronounced symptoms of Septoria blotch, collected on the observed field along its diagonal plane at equal distances and a certain time period (for example, during the counting process).

To collect the samples of affected plants, crop observations were implemented in areas mentioned in Table 1. All the samples were collected during their maturation phase, at the stage of milky-wax ripeness of plants (75–85 according to the Zadok’s scale). The leaves with typical external signs of Septoria blotch disease were picked. The collected material was herbarized and labeled (indicating the place and date of collecting, the phase, plant species and cultivar, information concerning disease symptoms, culture cultivation technologies, information regarding protection measures). Subsequently, the infectious samples of grain crops (leaves) of wheat, triticale and oats were analyzed in laboratory conditions to identify the species composition of Septoria blotch pathogens (Pyzhikova et al., 1989).

**Table 1.**
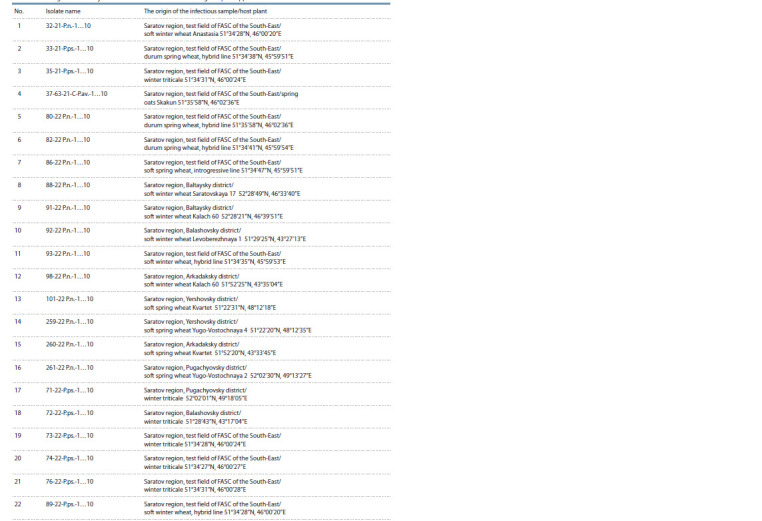
The origin of the analysed monoconidial isolates of Parastagonospora spp. in 2021–2022

Meteorological conditions of 2020–2022 in the region had a beneficial effect on the development of Septoria blotch causative agents of grain crops. According to the Saratov meteorological station data, at the beginning of the vegetation period in May, considering the three-year average, there was a 30.5 mm precipitation fallout. At moderate air temperatures, the hydrothermal coefficient (HTC) was quite high – 1.3. In June, the precipitation amount (34.5 mm) and HTC (0.55) decreased. In the middle of vegetation, in July, the situation improved significantly. Precipitation fallout was 97.2 mm, which is much higher than the norm, and the hydrothermal coefficient was high, showing 1.54. This contributed to the growth and development of agricultural plants and had a positive effect on the development of the phytopathogenic complex. In August, the precipitation level was low – 12.6 mm, on average, within the three-year period. Elevated air temperatures were noted – the number of days with a maximum air temperature above or equal to 30 °C was 17. The hydrothermal coefficient in this month was also extremely low – 0.2, indicating arid conditions.

The degree of damage of the infectious material selected for analysis varied from 30 to 40 %. The species of Z. tritici was isolated from all the samples of the infectious material subject to the study. It was possible to obtain monoconidial isolates of fungi of the Parastagonospora genus from some samples (see Table 1) (Pyzhikova et al., 1989).

The samples were analyzed in laboratory conditions to identify the species composition of Septoria blotch causative agents. The results of laboratory diagnostics of the specific affiliation of the pathogen were confirmed by the sequencing method using the equipment of the Collective Use Center “Genomic Technologies, Proteomics and Cell Biology” of the All-Russian Research Institute of Agricultural Meteorology

The analysis included 220 monoconidial isolates of the Parastagonospora genus, taken in the amount of 10 from each of the 22 infectious samples. To assess the resistance to Septoria blotch, 13 samples of winter wheat were used (Gostianum 237, Lutescens 230, Saratovskaya 8, Gubernia, Mironovskaya 808, Donskaya bezostaya, Saratovskaya 90, Zhemchuzhina Povolzhya, Saratovskaya 17, Kalach 60, Podruga, Anastasia, Sosedka) and seven samples of spring wheat (Favorit, Prokhorovka, Yugo-Vostochnaya 2, Saratovskaya 70, Saratovskaya 73, Belyanka, Lebyodushka). Screening was carried out on the test fields of the FASC of the South-East in the conditions of a natural infectious background in 2020–2022. A modified and supplemented Saari–Prescott scale was used (Kolomiets et al., 2017). All the cultivars were divided into five groups: RR – highly resistant (damage < 11 %); R – resistant (damage 11–20 %); MS – moderately susceptible (damage 21–40 %); S – susceptible (damage 41–70 %); HS – highly susceptible (damage 71–100 %).

The laboratory evaluation was implemented using isolated leaves as described by G.V. Pyzhikova and E.V. Karaseva (1985). Three inoculums of Z. tritici, P. nodorum (ToxA, Tox1, Tox3), P. pseudonodorum (ToxA, Tox1, Tox3) isolates were used. When inoculating plants under laboratory conditions, fungal isolates of the Saratov population obtained from the infectious material of 2022 were used: Z. tritici: 80-22-Z.t – durum spring wheat host, 80-22-Z.t – soft winter wheat, 95-22-Z.t – winter wheat; P. nodorum: 80-22-P.n. (Tox3) – durum spring wheat host, 101-22-P.n. (ToxA, Tox3) – soft spring wheat, 88-22-P.n. (Tox1, Tox3) – winter wheat; P. pseudonodorum: 72-22-P.ps. (Tox1, Tox3) – winter triticale, 89-22-P.ps. (Tox1). The presence of Tox genes in the genotypes of the Parastagonospora spp. fungi isolates used was detected for the first time and the results are presented in this work. An isolate of the Tambov population 82-21-P.ps., the genotype of which contains ToxA, was added to the infectious material of the P. pseudonodorum species. This isolate was obtained from the soft spring wheat Voronezhskaya 20 in 2021 (Zeleneva et al., 2022).

Fungal genomic DNA was isolated from a pure culture of monoconidial isolates obtained on potato glucose agar (CGA) using the standard CTAB method (Doyle J.J., Doyle J.L., 1990). The same method was chosen to extract DNA from young leaves of 13 winter and 7 soft spring wheat cultivars.

Amplification of genomic DNA was carried out in 25 μl of the reaction mixture (2 μl of genomic DNA (25 ng (permittable from 2 to 50 ng)), 1 μl of each primer (10 pM/μl) (Evrogen, Russia), 0.5 μl of dNTPs mix (10 mM, aqueous solution of dCTP, dGTP, dTTP and dATP) (TransGen, China), 0.55 μl MgCl2 (100 mM), 0.5 μl BioTaq DNA polymerase (5U, 5 U/ μl) (Dialat Ltd., Russia), 2.5 μl 10X PCR buffer, 17 μl ddH2O).

The amplified fragments were separated by electrophoresis in 1.5 % of agarose gel, in 1× TBE buffer (pH 8.2), the gel was stained with ethidium bromide. The DNA marker Step100 plus (Biolabmix, Russia) was used to assess the fragment size.

Screening of isolates of the Parastagonospora genus to detect the presence of effector genes: ToxA, Tox1 and Tox3 was performed using PCR. To obtain statistically valid results, the DNA of ten monoconidial isolates obtained from each infectious sample was analyzed (see Table 1). A total of 220 DNA samples were analyzed. The list of primers for PCR is presented in Table 2.

**Table 2.**
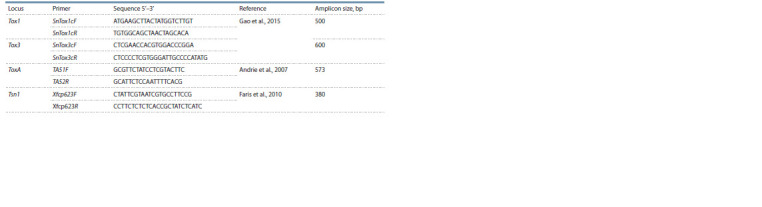
List of primers for PCR

Screening of genotypes of wheat cultivars aimed at revealing the presence of a dominant or recessive gene (Tsn1/tsn1) was put at work according to the method of using PCR with primer pairs Xfcp623(F)/Xfcp623(R) (Faris et al., 2010). The presence of the marker amplification product indicates the presence of the dominant allele of the Tsn1 gene (plant susceptibility to the PtrToxA fungal toxin protein), its absence signifies the presence of the recessive tsn1 allele (plant resistance to PtrToxA).

Statistical data processing was carried out using the computer program STATISTICA 12. The average damage of the leaf blade by Septoria blotch was calculated during the field assessment within the period of 2020 –2022, %; SD – standard deviation (Std. Dev.). The Q-Cochran criterion was taken to divide the studied wheat cultivars according to resistance/ susceptibility to three pathogens of Septoria blotch. This criterion was used to test a significant difference between phytopathological assessments of wheat cultivars.

## Results

**Fig. 1.**
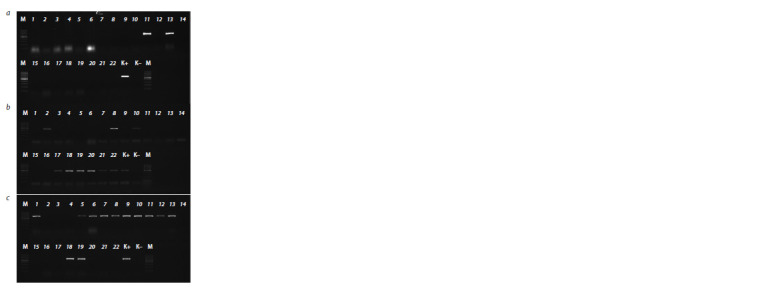
Electropherogram of amplification products obtained using markers specific to the ToxA, Tox1, and Tox3 genes of Parastagonospora spp. from the Saratov population a – ToxА, amplicon size 573 bp; b – Tox1, amplicon size 500 bp; c – Tox3, amplicon size 600 bp. М – Step100 plus marker (Biolabmix). The index numbers assigned to the samples correlate with the indexes in Table 1.

As a result of molecular screening, in the studied material (220 DNA probes obtained from 130 monoconidial isolates of P. nodorum, 80 P. pseudonodorum and 10 P. avenae isolates), both single genes encoding NEs and their combinations in one genotype were identified (Fig. 1, Supplementary Material 1)1.

Supplementary Materials are available in the online version of the paper: https://vavilov.elpub.ru/jour/manager/files/Suppl_Zeleneva_Engl_27_6.pdf


The ToxA gene was identified among monoconidial isolates of the P. nodorum species (93-22-P.n.-1…10) obtained from the leaves of a hybrid line of soft winter wheat from the experimental field of the FASC of the South-East and from the infectious material of soft spring wheat Kvartet (110-22- P.n.-1…10) from the Yershovsky district of the Saratov region (see Fig. 1, a).

As a result of molecular screening, the Tox1 gene was identified among the isolates obtained from four cultivars of wheat and five triticale cultivars. The presence of the gene was noted in P. nodorum isolates from the soft winter wheat cultivar of Saratovskaya 17 (88-22-P.n.-1…10) and Levoberezhnaya 1 (92-22-P.n.-1…10) from the Balashovsky district. The presence of the SnTox1 gene was marked in P. pseudonodorum monoconidial isolates taken from infectious material from hybrid lines of durum spring wheat (33-21-P.ps.-1…10) and soft winter wheat (89-22-P.ps.-1 ...10) from the experimental field of the FASC of the South-East, as well as winter triticale cultivars from the Pugachyovsky district (71-22-P.ps.-1 ... 10), Balashovsky district (72-22-P.ps.-1 ... 10) and from the experimental field of the FANC of the South-East (73-22-P.ps.- 1…10; 74-22-P.ps.-1…10; 76-22-P.ps.-1…10) (see Fig. 1, b).

The presence of the Tox3 gene was detected among isolates of the P. pseudonodorum species obtained from plant samples of winter triticale from the Balashovsky district and the experimental field of the FASC of the South-East (72-22- P.ps.-1…10, 73-22-P.ps.-1…10, respectively). The presence of the Tox3 gene was marked in isolates obtained from winter wheat cultivar Anastasia (32-21-P.n.-1...10), Saratovskaya 17 (88-22-P.n.-1...10), Kalach 60 (91-22-P.n.-1...10; 98-22-P.n.-1…10), Levoberezhnaya 1 (92-22-P.n.-1…10), hybrid line (93-22-P.n.-1…10); from spring wheat of hybrid lines (80-22-P.n.-1…10; 82-22-P.n.-1…10), introgressive line (86- 22-P.n.-1…10), from the Kvartet cultivar (101-22-P.n.- 1…10) (see Fig. 1, c).

In the course of three-year tests on a natural infectious background, cultivars showing resistance or weak susceptibility to Septoria blotch were detected (Table 3)

**Table 3.**
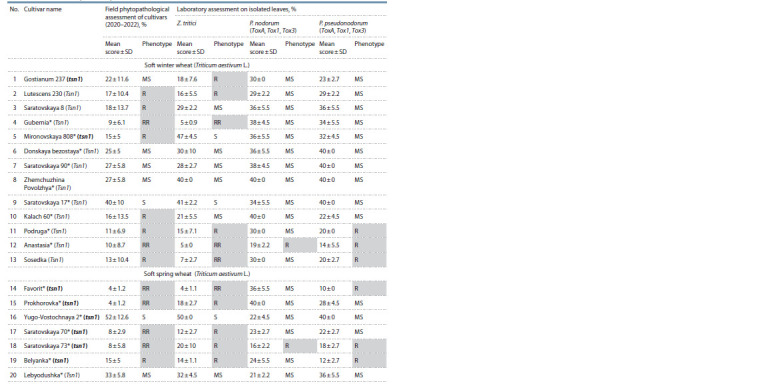
Damage intensity caused by leaf diseases in soft spring and winter wheat cultivars * The cultivar is admitted to cultivation in the territory of the Lower Volga region of the Russia Federation (region 8).

**Fig. 2.**
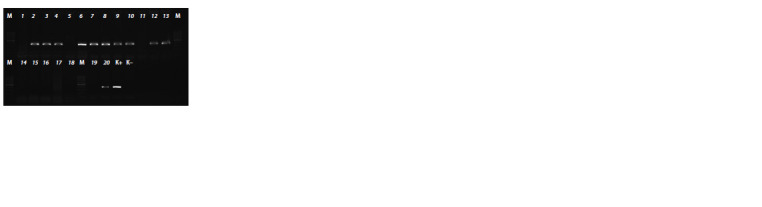
Electrophoregram of Tsn1 gene amplification products in soft winter and spring wheat cultivars (amplicon size 380 bp). The numbers indicated for the samples correspond to the list of cultivars in Table 3 (positive control (K+) – the cultivar of Glenlea, negative control (K–) – line 6B365).

Genotyping of wheat cultivars using a molecular marker was aimed at identifying carriers of genes that control sensitivity and resistance to the PtrToxA toxin. The Xfcp623 marker amplified a 380 bp. fragment associated with the Tsn1 gene sensitive to the PtrToxA toxin in 12 cultivars of soft winter wheat: Lutescens 230, Saratovskaya 8, Gubernia, Donskaya bezostaya, Saratovskaya 90, Zhemchuzhina Povolzhya, Saratovskaya 17, Kalach 60, Podruga, Anastasia, Sosedka and one cultivar of soft spring wheat, Lebyodushka. The genotypes of two cultivars of soft winter wheat: Gostianum 237 and Mironovskaya 808; six cultivars of soft spring whea: Favorit, Prokhorovka, Yugo-Vostochnaya 2, Saratovskaya 70, Saratovskaya 73, and Belyanka represent carriers of the recessive allele of the tsn1 gene and are protected against PtrToxA at their genetic level (see Fig. 2, Table 3).

A laboratory test of cultivars concerning the reaction to three pathogens of Septoria blotch, typical for the region (Z. tritici, P. nodorum (ToxA, Tox1, Tox3), P. pseudonodorum (ToxA, Tox1, Tox3)) was carried out. The infectious material of the regional populations of 2022 was used for inoculation. The results are presented in Table 3.

When wheat samples were infected with Z. tritici, the following cultivars performed well: Gubernia, Anastasia, Sosedka, Favorit. Their degree of damage did not exceed 7 % on average, they were included in the group of highly resistant cultivars (RR). The degree of damage by Z. tritici to Gostianum, Lutescens 230, Podruga, Prokhorovka, Saratovskaya 70, Saratovskaya 73, and Belyanka did not exceed 20 %, which made it possible to classify the cultivars as the members of the resistant group (R).

Two cultivars were confirmed to be resistant to P. nodorum: Anastasia and Saratovskaya 73. Six cultivars were confirmed to be resistant to P. pseudonodorum: Podruga, Anastasia, Sosedka, Favorit, Saratovskaya 73 and Belyanka.

When using the statistical method of correlation analysis, a weak direct relation was established between the indicators of the presence of the Tsn1 gene in the wheat cultivar genotype and the intensity of its damage caused by P. nodorum and P. pseudonodorum species containing the ToxA gene in isolates included in the inoculum (the correlation coefficient is 0.3 and 0.2, respectively).

A strong direct correlation was noted between the indicators of the overall degree of leaf blade damage caused by Septoria blotch in the field and the degree of damage to wheat samples caused by Z. tritici (0.8) and P. pseudonodorum (0.7) in the laboratory.

The indicators of the degree of damage to wheat cultivars of Z. tritici had a direct relation with the degree of P. pseudonodorum damage (0.77); P. nodorum and P. pseudonodorum (0.4); Z. tritici and P. nodorum (0.2).

The Q-Cochran criterion made it possible to divide the studied wheat cultivars into four groups based on the criterion of resistance to three pathogens: 1 – lack of resistance to Septoria blotch pathogens; 2 – resistance to one species; 3 – resistance to two species; 4 – resistance to three species of pathogens. The value of the coefficient Q = 36.35 with the significance level of p less than 0.009 indicates that the cultivars differed significantly from each other in terms of resistance/susceptibility to Septoria blotch pathogens of Z. tritici, P. nodorum, P. pseudonodorum. The test results are presented in Table 4 and Supplementary Material 2.

**Table 4.**
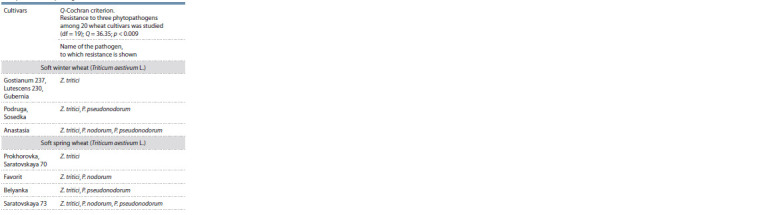
Non-parametric statistical analysis of indicators of phytopathological evaluation of cultivars to Septoria blotch pathogens

## Discussion

Septoria blotch represents a dangerous wheat disease; it is one of the most harmful in the fields of the Saratov region. In 2017, a strong epiphytoty of Septoria blotch was recorded on winter wheat crops (the damage was equal to 67 %). In 2018–2019, the intensity of Z. tritici damage did not exceed 25 %. Septoria blotch infection, which exceeded the threshold of 40 %, was noted in 2020 – 45 % and in 2021 – 41 % (Konkova et al., 2022).

The proposed study is one of the first in this region. It includes a comprehensive screening of area-specific and promising cultivars of soft winter and spring wheat, as well as molecular analysis that shows the presence of genes encoding NEs in plant pathogen populations and genes in plant genotypes that control disease resistance

In the course of the study carried out, it was shown that among the genotypes of the studied isolates of P. nodorum and P. pseudonodorum of the Saratov population, there was a wide representation of the Tox1 and Tox3 genes, while the ToxA gene was recorded only in isolates 93-22-P.n.-1…10 and 101-22-P.n.-1…10 of the P. nodorum species. The results obtained are consistent with foreign publications (Richards et al., 2022) reporting that the prevalence of the Tox267 and Tox1 genes is significantly higher than that of ToxA in the genotypes of P. nodorum populations that are territorially distant from Russian ones.

It is known that P. nodorum is a donor of the ToxA gene for Pyrenophora tritici-repentis; they share a common toxin, PtrToxA (Ciuffetti et al., 1997). Recently, the ToxA gene was identified in the fungus that causes wheat blotch – Bipolaris sorokiniana (McDonald et al., 2017; Friesen et al., 2018). This means that the cultivars of winter wheat Gostianum 237 (tsn1), Mironovskaya 808 (tsn1) and spring wheat Favorit (tsn1), Prokhorovka (tsn1), Yugo-Vostochnaya 2 (tsn1), Saratovskaya 70 (tsn1), Saratovskaya 73 (tsn1), Belyanka (tsn1) are protected from the ToxA gene toxin of four dangerous phytopathogens at once (P. tritici-repentis, P. nodorum, P. pseudonodorum, B. sorokiniana).

In the work of N.M. Kovalenko and colleagues (2022), it is possible to see the results of identification of the Tsn1/tsn1 allele using the Xfcp623 molecular marker in 35 cultivars of winter and 31 cultivars of spring wheat, included in the State Register of Breeding Achievements in 2018–2020 for the first time. Out of them, only 9 cultivars of winter and 4 cultivars of spring wheat carried Tsn1, which indicates susceptibility to PtrToxA, while the remaining cultivars have protection against the toxin at the genetic level. We consider this a great achievement of national selection. In the work of T.L. Friesen and colleagues (2018), it was mentioned that maintaining tsn1 in the genotypes of wheat cultivars admitted for selection not only provides a selective advantage over pathogens that currently carry ToxA, but may also exert selection pressure on newer or more suitable pathogens that acquire ToxA via horizontal transfer.

## Conclusion

Thus, using molecular markers, the identification of genes encoding NEs in two species called P. nodorum and P. pseudonodorum from populations of the Saratov region was carried out. In monoconidial isolates, both single Tox1, Tox3, and ToxA genes, as well as combinations of two genes in one genotype, were noted. The presence of a characteristic amplification product suggests the presence of two NEs genes, ToxA and Tox3, in P. nodorum monoconidial isolates 93-22-P.n.-1…10 and 101-22-P.n.-1…10; Tox1 and Tox3 in isolates 88-22-P.n.- 1…10, 92-22-P.n.-1…10. 72-22-P.ps.-1…10. One Tox1 gene in isolate genotypes 33-21-P.n.-1…10, 71-22-P.ps.-1…10, 89-22-P.ps.-1…10. Tox3 gene in genotypes 32-21-P.n.-1…10; 80-22-P.n.-1…10, 82-22-P.n.-1…10, 86-22-P.n.-1…10; 91- 22-P.n.-1…10; 98-22-P.n.-1…10 and 73-22-P.ps.-1…10.

A collection of 20 cultivars (16 area-specific and 4 promising) was studied to detect resistance/susceptibility to Septoria blotch pathogens in the experimental field of the FASC of the South-East in the period of 2020–2022, as well as in laboratory conditions. The cultivars underwent PCR screening showing the presence of a dominant or recessive gene (Tsn1/ tsn1), which controls sensitivity to the toxin of the fungus PtrToxA. For this reason, 11 cultivars with resistance to one, two or three types of phytopathogens (Z. tritici, P. nodorum, P. pseudonodorum) are of the greatest interest. These are the cultivars of the Saratov selection Anastasia (Tsn1), Belyanka (tsn1), Gostianum 237 (tsn1), Guberniya (Tsn1), Lutescens 230 (Tsn1), Podruga (Tsn1), Prokhorovka (tsn1), Saratovskaya 70 (tsn1), Saratovskaya 73 (tsn1), Sosedka (Tsn1) and Favorit (tsn1).

## Conflict of interest

The authors declare no conflict of interest.
